# Supplementary material for: Burnout and depression: A cross sectional study among health care workers providing HIV care during the COVID-19 pandemic in Malawi
Source: PLOS Glob Public Health. 2023 Sep 20;3(9):e0001887. doi: 10.1371/journal.pgph.0001887 (PMC10511108; doi:10.1371/journal.pgph.0001887)
Supplement: Attachment — Submitted filename: PGPH response to reviewer.docx [file pgph.0001887.s001.docx]

**Reviewer comments**

***Reviewer #1***

Thanks for the opportunity to review this paper by Phiri et al. The paper has described the prevalence and factors associated with burnout and depression among health care providers in Malawi. Overall, the paper reads well, however, I have few comments and suggestions as follows

**RESPONSE**: Thank you for the attentive and positive review.

1. It would be helpful for the authors to detail how the facilities were selected. There is a mention in the abstract that facilities were randomly selected – this is unclear? Was it proportional to HCW population size in the facilities?

**RESPONSE**: Facilities were purposively selected, by including facilities that had a high HIV burden and where COVID-19 greatly affected HIV service delivery. We have added a statement to clarify this in the Methods section (line 94-95).

2. It is unclear why the authors decided to select a few participants randomly at each of the participating facilities.

**RESPONSE**: An overall sample size was determined using a standard sample size formula for estimating proportions and based on the prevalence of burnout derived from a study that was conducted in Malawi in 2018.

Due to lack of data on the number of HCWs within different cadres at each of the 32 purposively selected facilities, which would be required to draw a sampling frame, we were not able to distribute this sample across the facilities proportionate to size, in order to have a self-weighted sample. Instead, the calculated sample size of 435 was equally distributed over the 32 health facilities, resulting in 14 HCWs required per facility. This is indeed a limitation and we have acknowledged this in lines 95-101.

3. The sample size estimation should have accounted for a design effect – given the utilization of multistage sampling approach.

**RESPONSE**: The multi-stage sampling approach used in this study has some limitations as explained in #2. This made it not possible to consider design effect during sample size calculation. As mentioned above, this limitation has been acknowledged in lines 96-102.

4. It is critical that the analysis to account for the survey design – i.e., the sampling weights, clustering and stratification at the facility level..

**RESPONSE**: All logistic regression models used in the analysis were adjusted for clustering at the facility level. We did this by using standard errors that allowed for intragroup correlation at facility level. A statement has been added to the methods section to highlight this in lines 153-154.

5. The analysis of data is unclear. First, it is unclear from the analysis of the data that this study had two main outcomes – the burnout and depression. These should be described separately, especially the multivariable logistic regression analysis. Second, the literature used to determine the factors included in the multivariable model should be cited (included).

**RESPONSE**: As advised, the methods section has been re-organized to explain the two main outcomes, burnout and depression, more clearly. References to relevant literature have been included. See lines 150-151

6. It is unclear whether the WHO SRQ was validated for use in Malawi.

**RESPONSE:** The WHO SRQ was validated in Malawi in 2008. We included this information in the manuscript line 130

Some additional minor comments:

1. The authors should consider standardizing the reporting of p values.

**RESPONSE:** This has been done in all our tables where p values are reported

2. Include ethics approval reference numbers.

**RESPONSE:** This has now been included in the manuscript, lines 125-126

(NHSRC REF # 19/08/2338), (UCLA IRB REF#21-000392)

3. Include the implications if the findings (potential interventions , not only screening) in the abstract conclusion section.

**RESPONSE:** we have included the suggestion the abstract lines 47-48

***Reviewer #2:***

This is an interesting and well-conducted study. I have a few suggestions to improve the manuscript’s quality and some concerns described below.

**RESPONSE:** Thank you for the attentive and positive review.

INTRODUCTION: Please include a brief summary of the HIV/AIDS epidemic in Malawi - prevalence, number of PLWHA under follow-up & receiving ART etc.

**RESPONSE:** We have included additional background information as suggested, in lines 65-67

COVID-19 context: This information belongs to your introduction section. Although the authors’ included the absolute number of COVID-19 cases, it would also be important to have the prevalence & mortality rate (if this data is available)

**RESPONSE**: We agree with your suggestion. We have moved the COVID-19 context section to the introduction and we have provided the prevalence and case fatality rates. See lines 72-78

METHODS: Burnout and depression are two mental health disorders that are frequently highly correlated… An analysis of confounding and interaction between the variables should be carried out, as well as a statistical collinearity assessment. I would advise the authors to include a collinearity diagnostic, evaluating the variance inflation factor (VIF) value between SRQ & MBI.

**RESPONSE**: In our analyses, the two outcomes of interest are depression and burnout. We used multivariable logistic regression models to evaluate the impact of socio-demographic factors on both depression and burnout. Since we did not use depression and burnout as explanatory variables, we do not believe that collinearity between the two variables is an issue, nor do we see a need to evaluate the variance inflation factor.

DISCUSSION, 6th paragraph: “Evidence-based treatment for depression, which is feasible in low-resource settings (…).” This statement is controversial. There is a huge treatment gap for mental disorders in low-and middle-income countries (LMIC). LMIC struggle with scarce resources, competing public health priorities, human resource shortages, low investment in mental health services, fragmented service delivery models etc. Those are just a few of the challenges faced by LMIC to properly evaluate the burden of mental disorders and allocate adequate funds/personnel. Please revise your statement.

**RESPONSE:** Thank you for this important comment. We have adapted the text in lines 262-265 of the discussion and added a reference.

DISCUSSION: Roughly 30% of study participants screened positive for depression and/or burnout. Undetected and untreated mental disorders are common in LMIC. One key question: Were healthcare workers screened positive for depression and/or burnout referred to additional mental health evaluation and possible treatment? Did they receive prompt mental health counselling after the survey? Did they have access to their screening results? The suicide rate among healthcare professionals during the COVID-19 pandemic was extremely high, especially among those with depression and/or experiencing burnout. What were additional ethical procedures in place for study participants identified as having those mental disorders? Please address this concern.

**RESPONSE:** HCWs received their screening results. HCWs who screened positive for depression and burnout were offered counseling by lay cadre staff and psycho-social counselors. Those with the highest scores on depression and burnout screening scales were referred to existing mental health clinics. We did not experience any suicides during the COVID-19 epidemic among our staff, when we had set up district-based mental health teams who proactively interacted with staff to detect and address anxiety and stress.

We added a sentence to the text in the Methods section: Data collection tools and measurement of outcomes, lines 121-122

This study was conducted almost two years ago. I’m curious to know if any knowledge translation and exchange and/or knowledge mobilization strategies were implemented until the submission date, March 2023. For instance, were the study results presented at the selected health facilities? Any brief report was sent to HCW, staff, health clinic directors etc.? During the COVID-19 pandemic, a vast amount of knowledge was created. However, only a fraction of it was effectively mobilized—shared, used and applied—to inform real-life situations. The lack of knowledge transfer or exchange contributes to a gap between research, policy and practice, hindering social innovation and slowing progress in many areas, including global mental health. This is a crucial concern everywhere. But in LMIC, resources are extremely limited. The results of studies such as the one described in this manuscript could inform new practices or (at least) highlight a hidden problem. Please discuss this concern.

**RESPONSE:** The study results were disseminated to relevant stakeholders including health care workers. In 2022, national HIV guidelines have introduced screening for depression and alcohol and substance abuse among ART clients. During the COVID-19 epidemic, our organization set up district-based mental health teams who proactively interacted with staff to detect and address anxiety and stress. However, no systematic approaches to screening for and management of burnout and depression among HCWs are in place currently. We agree that much more needs to be done for HCWs’ mental health and this is emphasized in the conclusion of the manuscript. We therefore did not change the text.
