# Supplementary material for: Burnout and depression: A cross sectional study among health care workers providing HIV care during the COVID-19 pandemic in Malawi
Source: PLOS Glob Public Health. 2023 Sep 20;3(9):e0001887. doi: 10.1371/journal.pgph.0001887 (PMC10511108; doi:10.1371/journal.pgph.0001887)
Supplement: Attachment — Submitted filename: HCW burnout PGPH response to reviewers.docx [file pgph.0001887.s002.docx]

**Reviewer comments**

***Reviewer #1***

1. Thanks for comprehensive responses to the comments. Note that the p- value should not be reported as 0.00 but rather as <0.01.

**RESPONSE**: Thank you for this observation. We have now changed all p-values that were reported as 0.00 as <0.01

***Reviewer #3: PGPH-D_22-01998***

2. The research questions required to be formed and justified in the introduction.

**RESPONSE**: We believe that the introduction explains and logically leads up to the research question, which is formulated in the last sentence of the introduction, lines 85-86

3. The reporting guidelines did not follow the STROBE guidelines, it is highly recommended to use.

**RESPONSE**: Our manuscript was adjusted to comply with the STROBE reporting guidelines for cross-sectional studies, see attached the completed STROBE checklist with relevant text from our manuscript.

4. The sample size estimation should have accounted for a design effect – given the utilization of multistage sampling approach.

**RESPONSE**: We acknowledge limitations with the multi-stage sampling approach used in this study. We used a standard sample size formula for estimating proportions to determine the overall sample size and this was based on the prevalence of burnout derived from a study that was conducted in Malawi in 2018. Due to lack of data on the number of HCWs within different cadres at each of the 32 purposively selected facilities, which would be required to draw a sampling frame, we were not able to distribute this sample across the facilities proportionate to size, in order to have a self-weighted sample. Instead, the calculated sample size of 435 was equally distributed over the 32 health facilities, resulting in 14 HCWs required per facility. This is indeed a limitation and we have acknowledged this in lines 96-102

5. Author did the logistic regression; did they check for confounding? Effect modification? Multi-collinearity?

**RESPONSE**: “All logistic regression models controlled for age, district and urban/rural residence as potential confounders.” This has been emphasized in lines 1154-157

6. Tools should be reported under subsection in the methods.

**RESPONSE**: Thank you for the observation, we have now presented the tools in a sub section. Refer to line 127

7. Report all the reliability and validity of the tools that were used.

**RESPONSE:** Both the World Health Organization (WHO) Self Report Questionnaire (SRQ) and the Maslach burnout inventory tool are reliable and validated tools in our setting and we have provided 3 references to underline this in lines 12-113.

8. “Descriptive statistics and multivariable logistic regression models were used to describe the prevalence of screening positive for depression and of screening positive for burnout.” How can the model describe the prevalence?

**RESPONSE:** We agree with the reviewer and have made corrections accordingly: “we used descriptive statistics to describe the prevalence of screening positive for depression and of screening positive for burnout” and “Multivariable logistic regression models were used to assess factors associated with positive screens for depression and burnout” as indicate in lines 1145-150

9. Did all assumptions of the regression analysis were met? No information about the assumption of logistic regression I can see.

**RESPONSE:** The basic assumptions applicable to logistic regression models were met: 1) observations are independent of each other, 2) there’s little or no multicollinearity among the independent variables. 3; the independent variables are linearly related to the log odds. We do not feel that it is important to state these conditions in the paper as that is not the standard practice for research articles in public health.

10. Before fitting the multivariable regression, the bivariable association requires to check.

**RESPONSE:** We have now added a column showing unadjusted ORs from bivariate analysis to the tables.

11. In multivariable regression, variable selection requires justification like check of confounding, interaction, and association in bivariate regression along with the support of the literature.

**RESPONSE:** We have rewritten the paragraph starting from line 151-157 to clarify these points.

12. After fitting the model, did they check the model fitness, such as sensitivity analysis, reporting BIC and AIC?

**RESPONSE:** In causal modelling, parameters used to assess goodness of fit or predictive accuracy are not a primary concern as the aim of the regression model isn't to describe the data or predict new observations but rather to make inferences about an underlying theory or hypothesis. We have added a citation that explains this.

13. The ROC curve with the AUC should be reported to make sure that the models predicted the outcome.

**RESPONSE**: In causal modelling, parameters used to assess goodness of fit or predictive accuracy are not a primary concern as the aim of the regression model isn't to describe the data or predict new observations but rather to make inferences about an underlying theory or hypothesis.

14. What is the statistical software used to require reporting?

**RESPONSE**: “All analyses were conducted using Stata v17.” Has been inserted.

15. “All multivariable logistic regression models used standard errors” is it the robust error variance? Require further explanation in the manuscript with reliable citations.

**RESPONSE:** We used cluster robust standard errors to allow for intra-cluster correlation. A citation has been included.

16. Give information on how the questionnaire was developed and encourage to provide it as a supplementary file.

**RESPONSE:** We used a modified Maslach burnout inventory tool and the world health organization self-report questionnaire for depression. The MBI tool is copy righted tool and we bought a license to use it. (Copyright ©1981, 2016 by Christina Maslach & Susan E. Jackson. All rights reserved in all media. Published by Mind Garden, Inc., [www.mindgarden.com](http://www.mindgarden.com)). A Chichewa version of the WHO self-report questionnaire was adapted for use by HCWs. The questionnaire was previously validated in Malawi by Stewart RC et al, 2008.

1. Authors may find the following articles helpful to correlate their findings with the following articles reported from South Asia:

https://doi.org/10.1371/journal.pgph.0000187

https://doi.org/10.1371/journal.pone.0274965

https://doi.org/10.1016/j.heliyon.2023.e13162

https://doi.org/10.3389/ijph.2022.1604769

https://doi.org/10.1111/inr.12802

https://doi.org/10.1186/s42269-022-00696-1

https://doi.org/10.1186/s43045-021-00103-x

https://doi.org/10.18332/popmed/142558

**RESPONSE:**

Thank you very much for referring us to the articles. We have gone through them and have seen some similarities in the findings for these articles and our findings. We have also cited the references to our text on line 239

STROBE Statement—Checklist of items that should be included in reports of ***cross-sectional studies***

|  | **Item No** | **Recommendation** | **Page No.** | **Relevant text in manuscript** |
| --- | --- | --- | --- | --- |
| **Title and abstract** | 1 | (*a*) Indicate the study’s design with a commonly used term in the title or the abstract | 1 | A cross-sectional study |
|  |  | (*b*) Provide in the abstract an informative and balanced summary of what was done and what was found | 2 | From April-May 2021, between the second and third COVID-19 waves in Malawi, we randomly selected HCWs from 32 purposively selected PEPFAR/USAID-supported health facilities for a cross-sectional survey. We screened for depression using the World Health Organization Self Report Questionnaire (positive screen: score≥8) and for burnout using the Maslach Burnout Inventory tool, (positive screen: moderate-high Emotional Exhaustion and/or moderate-high Depersonalization, and/or low-moderate Personal Accomplishment scores). Logistic regression models were used to evaluate factors associated with depression and burnout.  In analyses that controlled for age, district, and residence (rural/urban), we found that screening positive for depression was associated with expecting to be infected with COVID-19 in the next 12 months (aOR 2.7, 95%CI 1.3-5.5), and previously having a COVID-19 infection (aOR 2.58, 95CI 1.4-5.0). Screening positive for burnout was associated with being in the clinical cadre (aOR 1.86; 95% CI: 1.2-3.0) and having a positive depression screen (aOR 3.2; 95% CI: 1.9-5.4). |
| **Introduction** | | | | |
| Background/rationale | 2 | Explain the scientific background and rationale for the investigation being reported | 3-4 | Depression and burnout in particular threaten the wellbeing and effectiveness of HCWs [3, 4]. Evidence from before the COVID-19 pandemic shows high rates of psychological distress, anxiety, depression and burnout among HCWs in resource limited settings [5-8]. A systematic review from countries in Asia, South America, Europe and Africa, documented an overall prevalence of depression and depressive symptoms of 29% (range 21% to 43%) in the medical workforce [9]. Depression and burnout increase staff turnover, decrease quality of care provided to clients, and cause poor health outcomes among HCWs [10-12].  Research from before the COVID-19 pandemic found that burnout prevalence among HCWs in the sub-Saharan Africa region ranged from 40 to 80%, depending on country, type of HCW and the burnout measurement tool [17]. Workload may contribute to burnout [4]. In Malawi, the HIV prevalence among adults is 8.9%, and around 1,000,000 Malawians are living with HIV [18]. Of those, 88.3% are aware of their HIV-positive status and 97.9% are on antiretroviral therapy (ART) [18] This has created a huge care burden for HIV treatment clinics, which at the same time experience severe staff shortages, as only 28 nurses and two physicians are available per 100,000 population [19] . In such high-burden circumstances, HIV service providers may be at particularly high risk among HCWs to experience mental health problems.  For a variety of reasons, the COVID-19 pandemic may exacerbate mental health concerns among HCWs, including post-traumatic stress disorder, anxiety, depression and burnout [20].  At the time when the COVID-19 vaccine had just been rolled out in Malawi to HCWs as a priority population, we sought to assess the prevalence of depression and burnout and associated factors among HCWs working in HIV services during the COVID-19 pandemic in Malawi. |
| Objectives | 3 | State specific objectives, including any prespecified hypotheses | 4 | We sought to assess the prevalence of depression and burnout and associated factors among HCWs working in HIV services during the COVID-19 pandemic in Malawi. |
| **Methods** | | | | |
| Study design | 4 | Present key elements of study design early in the paper | 4 | We conducted a cross-sectional survey of 435 HCWs from 32 health facilities supported by Partners in Hope, a USAID/PEPFAR implementing partner that supports HIV/AIDS care and treatment in Malawi. |
| Setting | 5 | Describe the setting, locations, and relevant dates, including periods of recruitment, exposure, follow-up, and data collection | 5 | Selected sites included 25 public health facilities (6 urban and peri-urban district hospitals; 3 rural community hospitals; and 16 rural health centres) and 7 faith-based hospitals (3 urban and 4 rural) run by the Christian Health Association of Malawi. The health facilities were purposively selected across the three geographical regions of Malawi. We included facilities with a high HIV burden where COVID-19 greatly affected HIV service delivery.  From April-May 2021, between the second and third COVID-19 waves in Malawi, we invited up to 14 HCWs per facility to participate in an anonymous survey. |
| Participants | 6 | (*a*) Give the eligibility criteria, and the sources and methods of selection of participants | 5 | Eligibility criteria were: ≥18 years of age and providing outpatient HIV care during the three months preceding data collection.  We stratified HCWs into clinical and lay cadres, then randomly selected one individual per stratum and repeated this until up to 14 health care workers were invited for participation per facility. |
| Variables | 7 | Clearly define all outcomes, exposures, predictors, potential confounders, and effect modifiers. Give diagnostic criteria, if applicable | 6-7 | *Outcome 1*-Depression: We screened for depression using the World Health Organization Self Report Questionnaire (positive screen: score≥8). The WHO SRQ tool is comprised of 20 yes/no questions about physical, mental and emotional symptoms in the past 30 days. In order to capture experiences during the last COVID-19 wave, we extended this period to 3 months. “Yes” responses were assigned 1 point, and “no” responses 0 points; the responses were summed (possible range: 0-20), and the threshold for a positive screen for depression was set at ≥8,  *Outcome 2*-Burnout: The validated MBI tool has 22 questions across three domains of burnout: emotional exhaustion (EE), which measures exhaustion at work; depersonalization (DP), which measures negative or cynical feelings; and low personal accomplishment (PA), which measures feelings about one’s competencies. Each item asks how often respondents feel that way about their job, rated on a seven-point Likert scale (0-6). Sums were calculated for each of the three domains and scores were classified as low, moderate or high. Positive screen: moderate-high Emotional Exhaustion and/or moderate-high Depersonalization, and/or low-moderate Personal Accomplishment scores).  Multivariable logistic regression models were used to assess factors associated with positive screen for depression and burnout. We used multivariable logistic regression to investigated the association between burnout and depression |
| Data sources/ measurement | 8* | For each variable of interest, give sources of data and details of methods of assessment (measurement). Describe comparability of assessment methods if there is more than one group | 7 | Multivariable logistic regression models were used to assess factors associated with positive screens for depression and burnout. We used multivariable logistic regression to investigate the association between burnout and depression |
| Bias | 9 | Describe any efforts to address potential sources of bias | 17 | We used surveys that relied on a research assistant to ask each question and record the answer. As mentioned above, this may have caused measurement error due to social desirability bias, particularly for the MBI burnout scale, which is typically self-administered, and may have resulted in an underestimation of burnout. Our cross-sectional survey also cannot determine causality of the associations found. Results may have differed during COVID-19 waves, and we did not capture cumulative, longer-term COVID-19 effects. Due to lack of a priori data on the total number of HCWs within clinical and lay cadres at the selected 32 facilities that were needed to draw a sampling frame, we were unable to estimate a self-weighted sample that would consider multi-stage sampling. Alternatively, we used standard statistical inferencing assumptions, hence the generalization of our findings to the population level must be considered with caution. |
| Study size | 10 | Explain how the study size was arrived at | 5 | The sample size was determined using a standard sample size formula for estimating proportions, where we included the prevalence of burnout derived from a study that was conducted in Malawi in 2018 [11]. Due to lack of data on the number of HCWs within different cadres at each of the 32 purposively selected facilities, which would be required to draw a sampling frame, we were not able to distribute this sample across the facilities proportionate to size, in order to have a self-weighted sample. Instead, the calculated sample size of 435 was equally distributed over the 32 health facilities, resulting in 14 HCW required per facility. |
| Quantitative variables | 11 | Explain how quantitative variables were handled in the analyses. If applicable, describe which groupings were chosen and why | 7 | Multivariable logistic regression models were used to assess factors associated with positive screens for depression and burnout. We used multivariable logistic regression to investigated the association between burnout and depression |
| Statistical methods | 12 | (*a*) Describe all statistical methods, including those used to control for confounding | 7 | We used descriptive statistics to describe the prevalence of screening positive for depression and of screening positive for burnout. Burnout prevalence was determined on the basis of its individual constructs of EE, DP and PA. Multivariable logistic regression models were used to assess factors associated with positive screen for depression and burnout. Lastly, we used multivariable logistic regression to investigated the association between burnout and depression.  Regression models were fitted using a causal modelling approach [28] to investigate causal relationships between independent variables, selected based on assumptions drawn from literature, and the outcome variables. Independent variables of interest include COVID-19 experiences and risk perceptions, HCW cadres, marital status, years of work [29, 30]. Multivariable logistic regression models controlled for age, district, and residence (rural/urban) as potential confounders. All multivariable logistic regressions models used cluster robust standard errors that allowed for intragroup correlation at the facility level to control for clustering [31]. |
|  |  | (*b*) Describe any methods used to examine subgroups and interactions |  |  |
|  |  | (*c*) Explain how missing data were addressed |  |  |
|  |  | (*d*) If applicable, describe analytical methods taking account of sampling strategy |  |  |
|  |  | (*e*) Describe any sensitivity analyses |  |  |
| **Results** | | | | |
| Participants | 13* | (a) Report numbers of individuals at each stage of study—eg numbers potentially eligible, examined for eligibility, confirmed eligible, included in the study, completing follow-up, and analysed | 8&11 | We included 435 HCWs in the study.  We excluded data clerks, and 360 HCWs remained available for the burnout analyses. |
|  |  | (b) Give reasons for non-participation at each stage |  | Data clerks were excluded from all analysis on burnout as they do not have face to face interactions with patients. The burnout measurement considers provider patient interactions. |
|  |  | (c) Consider use of a flow diagram |  |  |
| Descriptive data | 14* | (a) Give characteristics of study participants (eg demographic, clinical, social) and information on exposures and potential confounders | 8-9 | We collected data from 435 HCWs see Table 1 |
|  |  | (b) Indicate number of participants with missing data for each variable of interest |  |  |
| Outcome data | 15* | Report numbers of outcome events or summary measures |  |  |
| Main results | 16 | (*a*) Give unadjusted estimates and, if applicable, confounder-adjusted estimates and their precision (eg, 95% confidence interval). Make clear which confounders were adjusted for and why they were included | 2 | In analyses that controlled for age, district, and residence (rural/urban), we found that screening positive for depression was associated with expecting to be infected with COVID-19 in the next 12 months (aOR 2.7, 95%CI 1.3-5.5), and previously having a COVID-19 infection (aOR 2.58, 95CI 1.4-5.0). Screening positive for burnout was associated with being in the clinical cadre (aOR 1.86; 95% CI: 1.2-3.0) and having a positive depression screen (aOR 3.2; 95% CI: 1.9-5.4). |
|  |  | (*b*) Report category boundaries when continuous variables were categorized |  |  |
|  |  | (*c*) If relevant, consider translating estimates of relative risk into absolute risk for a meaningful time period |  |  |
| Other analyses | 17 | Report other analyses done—eg analyses of subgroups and interactions, and sensitivity analyses |  |  |
| **Discussion** | | | | |
| Key results | 18 | Summarise key results with reference to study objectives | 14 | A year into the COVID-19 pandemic, after two waves of COVID-19 in Malawi and prior to the availability of widespread vaccination, we found that 28% of Malawian HCWs in HIV care screened positive for depression, 29% for burnout and 13% for both. A positive screen for depression was associated with reporting a previous COVID-19 infection, and with believing that one would get COVID-19 in the next 12 months. Screening positive for burnout was associated with being a clinical health worker (versus being a lay cadre staff). |
| Limitations | 19 | Discuss limitations of the study, taking into account sources of potential bias or imprecision. Discuss both direction and magnitude of any potential bias | 16 | We used surveys that relied on a research assistant to ask each question and record the answer. As mentioned above, this may have caused measurement error due to social desirability bias, particularly for the MBI burnout scale, which is typically self-administered, and may have resulted in an underestimation of burnout. Our cross-sectional survey also cannot determine causality of the associations found. Results may have differed during COVID-19 waves, and we did not capture cumulative, longer-term COVID-19 effects. Due to lack of a priori data on the total number of HCWs within clinical and lay cadres at the selected 32 facilities that were needed to draw a sampling frame, we were unable to estimate a self-weighted sample that would consider multi-stage sampling. Alternatively, we used standard statistical inferencing assumptions, hence the generalization of our findings to the population level must be considered with caution. |
| Interpretation | 20 | Give a cautious overall interpretation of results considering objectives, limitations, multiplicity of analyses, results from similar studies, and other relevant evidence | 17 | While HCWs who provide HIV care in Malawi screened positive for burnout and depression commonly, prevalence rates were not higher than reported before the COVID-19 pandemic. Our results reinforce the importance of screening HCWs for burnout and depression given the negative consequences for mental health and work performance. Appropriate and feasible interventions at the health system and individual level need to be introduced urgently to address burnout and depression among HCWs in our setting. |
| Generalisability | 21 | Discuss the generalisability (external validity) of the study results | 16 | We used surveys that relied on a research assistant to ask each question and record the answer. As mentioned above, this may have caused measurement error due to social desirability bias, particularly for the MBI burnout scale, which is typically self-administered, and may have resulted in an underestimation of burnout. Our cross-sectional survey also cannot determine causality of the associations found. Results may have differed during COVID-19 waves, and we did not capture cumulative, longer-term COVID-19 effects. Due to lack of a priori data on the total number of HCWs within clinical and lay cadres at the selected 32 facilities that were needed to draw a sampling frame, we were unable to estimate a self-weighted sample that would consider multi-stage sampling. Alternatively, we used standard statistical inferencing assumptions, hence the generalization of our findings to the population level must be considered with caution. |
| **Other information** | | | | |
| Funding | 22 | Give the source of funding and the role of the funders for the present study and, if applicable, for the original study on which the present article is based |  | “We are grateful for funding by PEPFAR through USAID that allowed Partners in Hope to collect the data from HCWs in its supported health facilities for this analysis.” |

*Give information separately for exposed and unexposed groups.

**Note:** An Explanation and Elaboration article discusses each checklist item and gives methodological background and published examples of transparent reporting. The STROBE checklist is best used in conjunction with this article (freely available on the Web sites of PLoS Medicine at http://www.plosmedicine.org/, Annals of Internal Medicine at http://www.annals.org/, and Epidemiology at http://www.epidem.com/). Information on the STROBE Initiative is available at www.strobe-statement.org.
